# Portuguese version of Bern Illegitimates Task Scale: adaptation and evidence of validity

**DOI:** 10.1186/s40359-023-01061-1

**Published:** 2023-01-26

**Authors:** Paula C. Neves, Cláudia Andrade, Rui Paixão, José Tomás da Silva

**Affiliations:** 1 Coimbra Education School, Research Group in Social and Human Sciences (NICSH), Polytechnic of Coimbra, Coimbra, Portugal; 2 Center for Interdisciplinary Studies, University of Coimbra (CEIS20), Coimbra, Portugal; 3 Center of Psychology, University of Porto, Porto, Portugal; 4 Centre for Social Studies, Faculty of Psychology and Educational Sciences, University of Coimbra, Coimbra, Portugal

**Keywords:** Bern Illegitimate Tasks Scale, Illegitimate tasks, Psychometric properties, Validity

## Abstract

**Background:**

A new element for job stress called Illegitimate Tasks has been investigated in recent years, along with stress as an offense to self-theory. Illegitimate tasks are those that are either needless or unrelated to the employee's role and can be categorized as unreasonable tasks and unnecessary tasks.

**Methods:**

This study aimed to adapt the Bern Illegitimate Tasks Scale to Portuguese and provide evidence of its validity, through a confirmatory factor analysis with a sample of 472 workers from different sectors.

**Results:**

The internal consistency, of the whole scale, measured by Cronbach’s alpha, was 0.923 and 0.902 and 0.928 for the unnecessary tasks and, for unreasonable tasks subscales respectively. The confirmatory analysis supported a two-factor model and showed good to very good indexes of fit (CFI = 0.985; TLI = 0.997; SRMR = 0.035; RMSEA = 0.171).

**Discussion:**

The Portuguese version of the Bern Illegitimate Tasks Scale presents very good psychometric properties for the intended measurement goals and can now be used in research with Portuguese speaking samples.

## Introduction

Illegitimate tasks is a concept that has received increasing attention from the literature of "stress-as-Offense-to-Self" [39, 40] that focus on the factors that generate stress in the workplace [3, 11, 21].

The foundation of the "Stress-as-Offense-to-Self" framework is the idea that people strive to preserve a positive self-image and that threats to this are frequently at the core of stressful experiences [19, 39, 40].

As such, threats to self-esteem can come through two ways: through personal self-esteem and/or through social self-esteem. Personal self-esteem concerns to one's assessment at any given moment of one's own aspirational standards of competence, reliability, honesty, and performance.

Stress to the personal self-esteem results from the individual's evaluation of his or her performance and behavior in relation to his or her own standards and aspiration. If one's own criteria for good performance are not met and therefore one's self-image does not correspond to an image of competence and reliability, the individual feels inadequate [39]. In this situation a threat to personal self-esteem identified as stress by insufficiency [39, 40] arise and can trigger emotions such as shame and guilt.

Self-esteem can also be threatened through social self-esteem “which refers to the degree to which one feels esteemed, acknowledged, and appreciated by others” [, p. 208].^40^

Experiencing disrespect from others may likewise degrade one's psychological well-being. Others' disregard can come across as disrespectful, endangering a person's feeling of self-worth or self-esteem. These circumstances are known as stress as disrespect [39, 40] and are a source of tension generation. The perception of disrespect can originate in interpersonal negative relationships (e.g., harassing), but can also be caused by working conditions such as task assignment.

The prominent role that work plays in many people's lives means that professional roles can become an integral part of one's identity [15]. Because people tend to value their professional identity [2] and often tend to define themselves by their work [35] conditions and actions that reinforce their professional identity contribute to increased self-esteem, while threats to that identity are perceived as stressful [8].

Assigning tasks that are not associated with the core of one's occupation or professional identity can be interpreted as a threat to professional identity and signal a lack of respect for the employee in question, can be perceived as identity stressors [22, 42] and considered as illegitimate tasks [39].

Illegitimate tasks (IT) are tasks assigned and performed by workers that do not fit within their role expectations [20, 41] as a professional and are therefore interpreted as an offense to their self [39, 40]. They are tasks that send a social message that the organization and the leader do not respect them (the employees) which will bring pressure and tension on the worker with implications on the perception of their legitimate professional identity and self-esteem [37, 39].

Illegitimate tasks can be perceived as either unnecessary or unreasonable [39]. Tasks that are unnecessary should not be done at all or could have been avoided if the work had been better organized. Unnecessary tasks are diverse and occupation-specific but may include, e.g., useless reporting and documenting, time-wasting meetings. A task might be considered unreasonable because it seems to be an unfair demand in relation to one's occupational role, or if it puts an employee in an awkward or difficult situation [41]. If so, they are perceived as directly disrespecting a person and as an attack on their identity [38].

Nonetheless, the perceived reasonableness of tasks is context dependent on which the task is performed [41] and the scope of one's definition of role tasks [24]. A task is not considered illegitimate if the subject accepts it or if it is performed on the subject's own initiative [38]. In other words, is not the task as such is illegitimate, but its social significance and its relation to the professional role.

In accordance with this, illegitimate tasks can be considered as a special type of role conflict, namely, a type of personality-role conflict [13].

Although Semmer et al. [41] recognize that IT can be regarded as a specific construct within the wider domain of justice, they also consider a distinctive construct since, unlike justice, it focuses on the tasks themselves and combines role-expectations and professional identity.

Illegitimate tasks are conceptualized as a two-dimensional construct with unnecessary and unreasonable tasks as subconstructs. Different studies confirmed that illegitimate tasks is best represented as a latent construct with two factors (unreasonable and unnecessary tasks) each with 4 items [26, 38].

Although both facets are related (but are not identical) and both are considered illegitimate, several studies used only one or another dimension separately and concluded they predicted different outcomes [26, 27].

Unreasonable tasks are more harmful to the individual, because, by their nature, are more self-relevant and person-specific than unnecessary tasks [12]. Unreasonable tasks are more salient to the employee and have a deeper impact on the psyche, and thus are more strongly tied to negative emotional reactions [33, 36], such, exhaustion, and intention to leave [4]. Unnecessary tasks create unnecessary effort or serve no purpose and have been shown to be an harmful job stressors resulting in negative well-being and motivational outcomes [9, 26].

Overall, IT aggrieve employees’ professional identities and cause pressure and stress, constituting a stressor that hinders their identities [39].

Research show that IT are positively related with work–family conflict [43], turnover intention [4, 43] and negatively related to job satisfaction [3] and counterproductive work behavior [41, 44].

At individual level research also found a positive relationship between IT, and stress and psychological strain [3, 38], anger [9, 44] and resentment [25, 38] and negatively related to satisfaction with work performance [3].

At present, the 8-item Bern Illegitimate Tasks Scale (BITS) [19, 38] is the most widely used in related research on illegitimate tasks. Existing studies have obtained good results in the reliability and validity test of BITS scale in different cultural contexts [9, 22, 34], which indicates that the two-dimensional structure of illegitimate tasks has cross-cultural applicability.

Although IT performs as a task related stressor [12, 22, 39], to our knowledge, there is no research carried out in Portugal that address this construct.

Despite the contribution that the studies have made to Illegitimate Task Scale research, more research efforts are needed, especially in Portuguese–speaking countries which do not have, so far, standardized procedures to measure the construct. It is a well-known fact that “the adaptation of psychological instruments is a complex task that requires careful planning regarding its content maintenance, psychometric properties, and general validity for the intended population” [, p. 423]. This study intended to generate some essential information concerning the content adequacy of the translated BITS version, and about some of its measurement properties, mainly those regarding score reliability (e.g., internal consistency) and validity (construct-structural validity). Acknowledging that the validation process of a psychological procedure never truly ends, in this preliminary essay of the Portuguese European BITS, the choice was to focus firstly on score reliability to the extent to which the observed score(s) reflects the true (latent) score(s) and secondly, it aimed to further work on the validity process by ascertaining if the structural relations (e.g., measurement model) of the original BITS would also fit the Portuguese data. Achieving success on this first stage will open the door to future research onto the validity process in other domains (e.g., criterion-related validity).^6^

## Methodology

### Sample

The sample for this study consists of 472 workers from different professional sectors.

The mean age of the total sample is 43.69 years (*SD* = 11.41), of which 350 (74%) are men and 122 (25.8%) are women. Of these, 228 (48.2%) work in the Public Sector, 219 (46.3%) in the Private Sector, and 25 (5.3%) are self-employed. Regarding working hours, 290 (61.3%) work fixed hours, 111 (23.5%) work in shifts, and 55 (11.6%) have flexible hours. As for the level of education, 14 (3%) have elementary school education; 111 (23.5%) have secondary or vocational education, and most of the subjects 347 (73.4%) have higher education.

### Bern Illegitimate Task Scale (BITS)

The BITS [19, 41] is a self-report measure composed of 8 items organized in two dimensions: unreasonable tasks and unnecessary tasks. The items are answered on a Likert scale from 1 to 5 (from “never” to “frequently”). The first version [19] determined the two-dimensional structure of illegitimate tasks and their measurement items through and exploratory and confirmatory factor analysis. Among them, there are 4 items measuring unreasonable tasks and 5 items measuring unnecessary tasks. However, Semmer et al. [38, 41], in a later version, eliminated one of the unnecessary task dimension items, and the scale now consists of 8 items, 4 items for unreasonable tasks and 4 to unnecessary tasks. This new version of the 8-item BITS scale is the most widely used in IT research and will be the one to be adapted into Portuguese.

### Translation

The adaptation of psychological instruments from the source language into the target language is a complex task that needs to follow very strict guidelines (e.g., [10, 14, 16, 18]). During this process, one must provide both the evidence of the semantic equivalence of the items and the adequate psychometric properties of the new version of the instrument (e.g., [6]). In general, the literature specifies that instrument adaptation entails several essential stages, namely (1) instrument translation from the source language into the target language, (2) synthesis of the translated versions, (3) analysis of the synthesized version by expert judges, (4) back translation, and (5) a pilot study [6]. The translation of BITS into European Portuguese was done considering the above-mentioned procedures and during this process great care was taken to ensure that the final version was both suitable for the new context and also consistent with the original version. More specifically, initially two independent bilingual translators were charged to adapt the items into the Portuguese. After the process of instrument translation from English into the Portuguese was finished both translated versions were translated by another independent translator and subsequently both versions were summarized and synthesized, and a consensual version was produced. The final English version thus obtained was later compared to the original and great similarities in the semantic content were verified, but nonetheless the research team also decided that altering the structure of item stems would improve the clarity of the content and its suitability to the target population (that is, all discourse in the Portuguese version is made in form of statements and not questions). After a final consensus was reached by the intervening parts in the process, a final formulation was submitted to a pre-test [5]. The results of the pre-test suggested the appropriateness of items regarding their meaning and difficulty to the target population.

### Procedure

This study was conducted with a non-probability (convenience) sample consisting of professionals from different economic sectors. Data collection followed a snowball methodology using an anonymous online data collection server. The questionnaire was administered at a single time point. Demographic information was also collected and included age, gender, professional occupation, work schedule and educational level. Participation was voluntary, and all participants were assured of both identity and response confidentiality as participants was anonymous within the dataset. The given informed consent was a required to start the questionnaire. To participate in the study having a regular professional occupation was a set as a mandatory criterion. Institutional Ethics review board approval was obtained to complete this study (Nº25_CEIPC_2022).

### Statistical analysis

Items’ psychometric properties were initially assessed using descriptive statistics [mean, Standard Deviation (*SD*), skewness (*Sk*), and kurtosis (*Ku*)]. After that, a Confirmatory Factor Analysis (CFA) of the measuring instrument was performed. The CFA is most appropriately conducted with fully implemented assessment devices that have already demonstrated acceptable factorial validity. The rationale for CFA procedures in the present case is based on evidence provided by Jacobshagen [19], and replicated studies by Semmer et al. [38, 41]. Although, maximum likelihood (ML) is usually the preferred method of estimation in CFA analyses, an important assumption underlying this estimation procedure is that the scale of observed variables is continuous, and admittedly the data provided by the BITS, technically speaking, represent categorical data (e.g., Likert-scaled items). Although, for many years, researchers have tended to treat categorical data as if they were continuous, now we do have well-developed strategies for addressing the categorical nature of the data [28, 29]. Methodologists have developed several different approaches to the modelling and testing of categorical data and, particularly, ordered categorical data, namely via three primary estimators: unweight least squares (ULS), weight least squares (WLS) and diagonally weight least squares (DWLS). Further work, subsequently showed that corrections to the means and/or variances provided better (more robust) estimates of the data, and Brown [7] argued that the weighed least squares means and variances (WLSMV) estimator performs best in CFA modelling of categorical data. Following Brown [7] the WLSMV estimator developed by Muthén et al. [30] was used on the CFA of the BITS data. The CFA was performed with the M*plus* Version 8.4 [31]. The indexes used to appraise model fit were the following: the comparative fit index (CFI), the Tucker–Lewis index (TLI), the root mean square error of approximation (RMSEA), and the standardized root mean square residual (SRMR). According to Hu and Bentler [17], the indexes should be (for CFI and TLI) > 0.90 (preferably > 0.95) and for the RMSEA and SRMR < 0.08.

## Results

All items have a range (1–5) and the mean ranges from 2.71 (*SD* = 1.28) in IT 7 and 3.74 (*SD* = 1.15) in IT3. The skewness ranged from − 0.633 to 0.281, and the kurtosis ranged from − 1.066 to − 0.388. Accordingly, the items scores, univariately speaking, follow an approximately normal distribution for the studied population since their distributional properties, judging from the *Sk* and *Ku* values [23] are suggestive of appropriate psychometric sensitivity. The descriptive statistics of the items are shown in Table 1. Although, all skewness values are below ± 1, several items showed absolute kurtosis coefficients above 1, and the multivariate kurtosis of 21.15 (critical ratio, c.r. = 18.17) suggests a significant deviation of the normality assumption and further argues against the use of the ML estimation on the present data versus the use of more robust estimators (e.g., WLSMV).

**Table 1:** Descriptive statistics of the BITS items

	Range	Mean (*SD*)	Kurtosis	Skewness
IT1	1–5	3.43 (1.25)	− 0.739	− 0.465
IT2	1–5	3.46 (1.24)	− 0.679	− 0.467
IT3	1–5	3.74 (1.15)	− 0.383	− 0.633
IT4	1–5	3.70 (1.22)	− 0.579	− 0.633
IT5	1–5	3.43 (1.37)	− 1.065	− 0.427
IT6	1–5	3.10 (1.31)	− 1.048	− 0.119
IT7	1–5	2.71 (1.28)	− 0.937	0.281
IT8	1–5	2.88 (1.32)	− 1.066	0.151

### Confirmatory factor analysis

The model used in the CFA was determined by the original model proposed by Jacobshagen [19] and later confirmed by Semmer et al. [38, 41]. According to these authors the responses to the BITS can be explained by two correlated factors (unnecessary tasks and unreasonable tasks) and each item has a non-zero loading on the factor it was designed to measure, and zero loadings on the other factor. Additionally, the residuals associated with each item are assumed to be uncorrelated.

The model test was based on the covariance matrix and used the WLSMV estimation as implemented in M*plus* 8.4 [31].

Fit indices for the model were generally acceptable: ${\chi }^{2}$ (19) = 280.42, *p* < 0.001, *n* = 472; CFI = 0.985; TLI = 0.977; SRMR = 0.035; RMSEA = 0.171; *p* (RMSEA ≤ 0.05) < 0.001; 90% CI (0.153; 0.189). All factor loadings were statistically significant (Table 2).

**Table 2:** WLSMV unstandardized factor loadings (critical ratios), and corrected item-total correlation

	F 1	F 2	Corrected item-total correlation
Do you have work tasks to take care of, which keep you wondering if../**No meu trabalho tenho tarefas a realizar que me mantêm a pensar se**…			
IT1- … they have to be done at all?**/… têm de ser feitas de todo?**	1.000		0.689
IT2- …they make sense at all?/…**fazem algum sentido?**	1.006 (43.07)		0.698
IT3-… they would not exist (or could be done with less effort) if it were organized differently?/… **não existiriam (ou poderiam ser feitas com menos esforço) se estivessem organizadas de forma diferente?**	0.976 (50.19)		0.705
IT4 … they just exist because some people simply demand it this way?/… **só existem porque algumas pessoas simplesmente o exigem desta forma?**	1.024 (51.54)		0.756
Do you have work tasks to take care of which you believe **No meu trabalho tenho tarefas a cumprir que acredito**			
IT5-… should be done by someone else?/…**devem ser feitas por outra pessoa**		1.000	0.687
IT6- … are going too far which should not be expected from you/…**estão a ir longe demais e que não deveriam esperar que eu as realizasse**		1.141 (44.78)	0.832
IT7- … put you into an awkward position?/…**estão a colocar-me numa posição incómoda**		1.108 (47.05)	0.746
IT8 -… are unfair that you have to deal with them?/… **é injusto que tenha de lidar com elas**		1.160 (46.36)	0.807

**Fig. 1:**
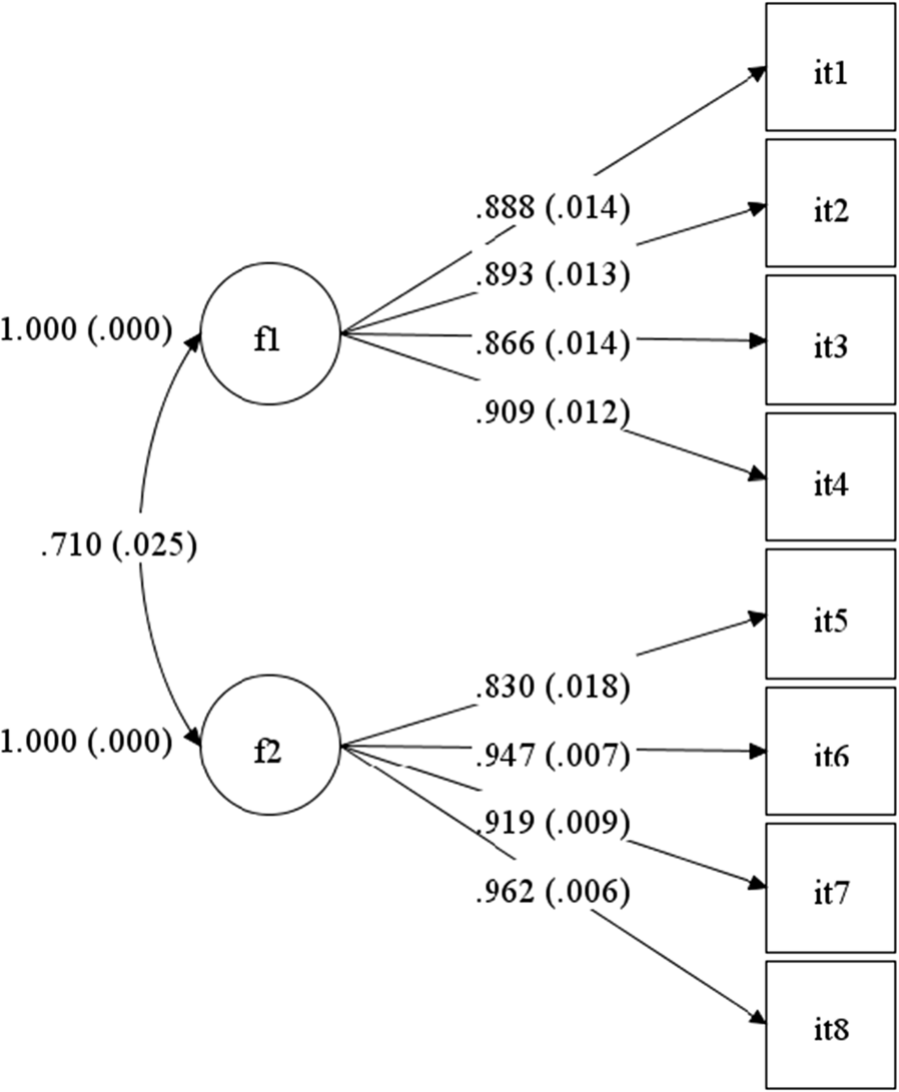
Illegitimate task construct path model

Standardized parameter estimates for the model are presented in Fig. 1. As said all model parameters were significant (*p* < 0.01) and explained substantial amounts of item variance (*R*^2^ = 0.79 to 0.93). Figure 1 also presents the disattenuated correlation between the factors (*r* = 0.71, c.r. = 27.98, *p* < 0.001).

The reliability of all scale items was calculated by Cronbach’s ordinal α = 0.923. Each of the items of the scale had a corrected item-total correlation of more than 0.68 (see Table 2). Moreover, the reliability for each subscale was Cronbach’s ordinal α = 0.902 for unnecessary tasks (IT1–IT4) and Cronbach’s ordinal α = 0.928 for unreasonable tasks (IT5–IT8).

Faced with results such as those obtained with the present CFA, authors may well try to engage in a post hoc specification search to improve the fit of the model (for example, the RMSEA value shows that there is some room for improvement), but given that all the estimated parameters are significant and that three (out of five) indices showed very good fit (e.g., CFI, TLI, and SRMR) we decided not doing any further changes to the model, because (a) of the hazards of empirically generated modifications, (b) and also because the fit measures derived from our study are very similar (and some cases even better) than those reported by Semmer et al. [38] replication study of the BITS measure (Table 3).

**Table 3:** Fit indices: Portuguese versus Semmer et al. [38] analysis

Model	*χ*^2^	*Df*	CFI	TLI	RMSEA	SRMR
Portuguese version	280.42	19	0.985	997	0.171	0.035
Semmer et al. [38]	85.81	29	0.935	0.898	0.102	0.050

## Discussion

The current study aimed to analyze the psychometric qualities of the BITS [19, 38] in order to address the lack of measures focused on the practices of illegitimate tasks in Portuguese that facilitate research in organizational settings. Results showed a 2-factor structure (unnecessary tasks and unreasolable taks) with an adequate fit. The translation of the scale was performed according to the procedures suggested by Figueiredo and Lemkau [10], and Hill and Hill [16].

Our results are consistent with previous research carried out in different cultures [22, 34] in terms of the reliability indices and factor structure. The Portuguese version showed good internal consistency, both on the global scale (Cronbach’s ordinal α = 0.923) as for each of the subscales, unnecessary tasks (Cronbach’s ordinal α = 0.902.), unreasonable tasks (Cronbach’s ordinal α = 0.928).

The CFA revealed similar results compared to those presented by [38] and indicated that the model fit adequately to the sample (Table 3).

Overall, the results suggest that the BITS adapted to the Portuguese context can be considered a valid and reliable tool for researchers and practitioners working in the organizational field. Since this measure contains only eight items is, therefore, a short and practical instrument, which offers an understanding of the practice of illegitimate tasks that has been shown to have important implications for employee’s well-being.

In fact, research consistently found that illegitimate tasks are identified as work stressors related with several individual negative outcomes, like job burnout and irritability [38], job dissatisfaction [9], lower intrinsic motivation at work [32] and low self-esteem [38]. It also presents negative organizational effects such as, increased turnover intentions [1], decrease in employees’ performance and proactive work behavior [22]. Altogether, the results of the previously mentioned studies claim the attention for the importance of not only have a deeper understanding of the concept, but also have adequate instruments to evaluate it in different cultures. Additionally, the identification of tasks that the employees consider illegitimate can provide important information for managerial practices. For instance, it could sign that either the employees need work relocation because they do not recognize that the tasks they are assigned are legitimate to their role [25] or the job itself is not well designed [11, 21]. Moreover, uncovering tasks that are considered illegitimate by the employees but considered relevant by the managers because they cannot be avoided in organizations, allows to promote better communication strategies focusing in providing information that can influence employee’s perception of their legitimacy.

### Limitations

Despite the findings, it is important to acknowledge some limitations of the present study and to highlight possible paths for future research. First, a convenience sample was used, so results should be generalized with caution. Thus, future research could use a representative and diverse sample from specific professional cohorts to have a deeper analysis of the results that were found in the current study. Second, the use of self–report data that may increase the probability of incurring common method variance. Therefore, it would be interesting for future research to move toward an external measure of illegitimate task.

Another limitation is that criteria validity studies were not performed. The objective of this study, being the first carried out in Portugal, was primarily to test the structural validity of the instrument and to corroborate if the two previously identified factors of the BITS were also valid and reliable constructs in the Portuguese population. Thus, further studies are needed to study more thoroughly other facets of validity, such as the BITS scores criterion validity.

## Conclusion

The BITS [19, 41] is a self-report measure composed of 8 items developed to measure a new stressor concept called Illegitimate Tasks. The initial structure proposed by the authors, single construct with the two first order factors (unnecessary tasks and unreasonable tasks) which are related, yet distinct facets, was confirmed in a sample of the Portuguese population.

Well-being continues to be crucial theme to organizational research and practice, given its importance to individual workers, as well as to the organizational performance. The availability of a short and practical instrument which can offer a diagnosis of the practice of illegitimate tasks within the organizational can help monitoring these activities to promote work interventions. Overall, the use of BIT in organizational contexts can facilitate both research and interventions for better organizational functioning.

## Data Availability

The data that support the findings of this study are available upon reasonable request to the authors.

## Abbreviations

BITS: Bern Illegitimate Task Scale
IT: Illegitimate tasks
